# Commercial AI for CT lung cancer screening: product capabilities, coverage of nodule management tasks and supporting evidence

**DOI:** 10.1007/s00330-026-12580-x

**Published:** 2026-05-07

**Authors:** Noa Antonissen, Steven Schalekamp, Horst K. Hahn, Kicky G. van Leeuwen, Colin Jacobs

**Affiliations:** 1 Diagnostic Image Analysis Group, Department of Medical Imaging, Radboud University Medical Center, Radboudumc, The Netherlands; 2 University of Bremen and Fraunhofer MEVIS, Bremen, Germany; 3 Romion Health, Utrecht, The Netherlands; 4 Health AI Register, Utrecht, The Netherlands

**Keywords:** Artificial intelligence, Radiology, Device approval, Evidence-based practice, Lung neoplasms

## Abstract

**Objectives:**

To characterize the capabilities of CE-marked AI products for lung nodule analysis in lung cancer screening (LCS), quantify their coverage of tasks defined in nodule management recommendations, and assess their peer-reviewed evidence.

**Materials and methods:**

Six core tasks in LCS (nodule detection, classification, measurement, growth assessment, malignancy risk estimation, and structured management) were derived from 4 nodule management recommendations: Lung-RADS 2022, British Thoracic Society (BTS) guidelines, European Union Position Statement (EUPS), and European Society of Thoracic Imaging (ESTI). Products were identified through www.healthairegister.com. Vendors confirmed capabilities using a standardized questionnaire; public documentation supplemented non-responders. Task coverage was calculated as the percentage of functional overlap (0–100%) per recommendation. Peer-reviewed evidence was evaluated using a six-level efficacy framework and assessed for study characteristics.

**Results:**

In total, 16 products from 16 vendors were included; 10 vendors completed questionnaires. Analysis showed that 14 products detect and measure solid and subsolid nodules, 12 support growth assessment, and 9 provide malignancy risk estimation (PanCan in 5, AI-based scores in 4). No product provides support for endobronchial or cystic lesions. High task coverage (> 75%) was observed in 10 products for EUPS and 4 for BTS, whereas no product achieved high coverage for Lung-RADS or ESTI. Overall, 60 peer-reviewed studies were identified; 7% were prospective and evidence clustered at lower efficacy levels: 70% assessed diagnostic accuracy, while none reported patient outcomes or societal impact.

**Conclusion:**

Numerous CE-certified AI products could support CT-based lung cancer screening, but gaps in task coverage and predominantly lower-level evidence necessitate cautious, monitored implementation.

**Key Points:**

***Question*** *Do commercially available AI products for lung nodule analysis functionally cover international nodule management recommendation-defined tasks, and what peer-reviewed clinical evidence supports them?*

***Findings**** AI products support standard nodule detection and measurement in line with management recommendations but lack support for endobronchial or cystic lesions and high-level clinical evidence*.

***Clinical relevance**** CE-marked AI products can assist radiologists with core lung cancer screening tasks, but capability gaps exist. Limited high-level clinical evidence complicates integrating AI into guidelines, securing reimbursement, and formulating recommendations for its use in lung cancer screening programs*.

**Graphical Abstract:**

**Figure:**
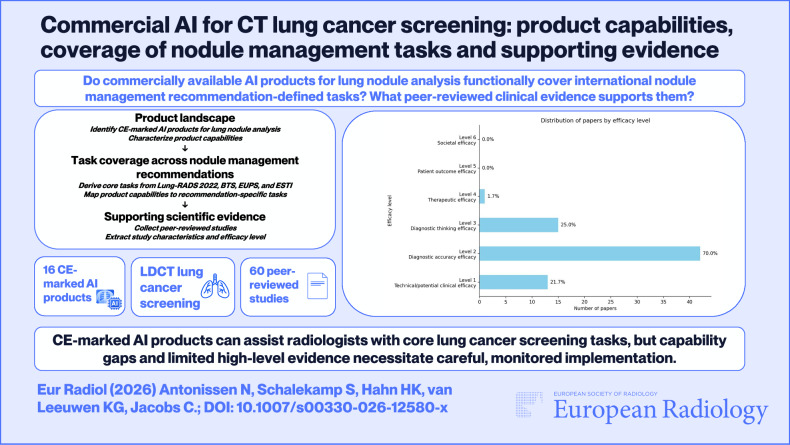

## Introduction

Lung cancer remains the leading cause of cancer-related mortality worldwide [1]. Early detection with low-dose computed tomography (LDCT) reduces mortality, as shown by the National Lung Screening Trial in the United States and the NELSON trial in Europe [2, 3]. Based on this evidence, LDCT screening has been adopted nationally in the United States and is being introduced across Europe, following the 2022 EU Council Recommendation inviting Member States to explore the feasibility and effectiveness of LDCT screening of individuals at high risk for lung cancer [4, 5]. Nevertheless, uptake within Europe remains heterogeneous, reflecting practical challenges in scaling lung cancer screening (LCS).

Key challenges include substantial reporting workload, inter-observer variability in pulmonary nodule assessment, and the need for efficient, standardized pathways [6]. Artificial intelligence (AI) has been proposed to alleviate the burden on radiologists by supporting or automating interpretation and reporting [7, 8]. Numerous Conformité Européenne (CE)-marked AI products for lung nodule analysis are now available [9] and positioned to assist LCS, yet uncertainty remains about their readiness for routine use, highlighting the need for clearly defined quality-assurance metrics [10].

Real-world implementation has already begun in several countries, offering early insights into AI integration. The United Kingdom’s Targeted Lung Health Check program employs AI as a second or concurrent reader to support radiologists [11]. Germany’s 2024 screening regulation mandates computer-assisted detection and volumetry for all screening LDCTs [12]. These implementations underscore both the momentum toward AI adoption and the urgent need to systematically evaluate which AI tools can meet clinical requirements and what evidence supports their deployment.

Lung cancer screening programs rely on established nodule management recommendations involving nodule detection, classification, measurement, growth assessment, risk stratification, and clinical management. These recommendations define the tasks where AI products can provide assistance. While national management protocols may differ, many are informed by widely used standards such as the Lung CT Screening Reporting and Data System (Lung-RADS) [13], the British Thoracic Society (BTS) pulmonary nodule guidelines [14], and the European Union Position Statement (EUPS) recommendations [15].

This study systematically evaluates CE-marked AI products for lung nodule analysis by characterizing their capabilities, assessing their coverage of tasks defined by nodule management recommendations, and classifying the available clinical evidence using a hierarchical efficacy model. Our goal is to identify where current AI products meet clinical requirements, reveal capability gaps, and characterize the strength of evidence supporting each product, thereby informing clinical adoption and priorities for future development.

## Materials and methods

### Selection of nodule management recommendations and task domains

To establish a reference for evaluating capabilities of commercial AI products for lung nodule assessment, we reviewed four major nodule management recommendations for screening: the Lung CT Screening Reporting and Data System (Lung-RADS) version 2022 [13], the British Thoracic Society (BTS) pulmonary nodule guidelines [14] and the European Union Position Statement on Lung Cancer Screening (EUPS) recommendations [15]. Additionally, we included the newly established European Society of Thoracic Imaging (ESTI) nodule management recommendations [16] as it is likely to shape future screening protocols. These recommendations were analyzed to identify recurring clinical tasks where AI support is valuable, yielding six prespecified core domains: nodule detection, classification, measurement, growth assessment, malignancy risk estimation, and clinical management recommendations.

### Selection of AI products

A systematic search was conducted to identify commercially available AI products developed for lung nodule analysis. Inclusion criteria required that products held CE certification for clinical use within Europe, were primarily designed for lung nodule analysis, and were available on the European market as of January 1, 2025. Eligible products were identified through the Health AI Register [9]. Products focusing primarily on general thoracic pathology or incidental findings, without dedicated lung nodule analysis, were excluded.

### Capability mapping and task coverage scoring

Information on the certified capabilities of each AI solution was initially extracted from publicly available sources, including the Health AI Register and vendor websites. This data was used to prefill a standardized questionnaire covering all predefined tasks and subtasks (see Appendix E1). The prefilled questionnaires were distributed in July 2025 to the identified vendors, who were invited to verify or modify the information. Since the ESTI nodule management recommendations were only published on July 1, 2025, we did not query whether products already provided ESTI-based management recommendations. Vendors were informed that, in the absence of a response, the analysis would proceed using publicly available data.

For each solution, product capabilities were mapped to the prespecified items and coded as supported, partially supported, not supported, or unverified. “Support” indicates clear evidence of the function in public documentation and/or vendor confirmation. “Partially supported” indicates incomplete functionality for a specified nodule type, where only some capabilities (detection, classification, or measurement) are certified rather than the complete set of functions. “Not supported” indicates explicit absence. “Unverified” indicates that public sources suggested the function, but evidence was incomplete or ambiguous, and the vendor did not confirm it.

To assess, across Lung-RADS, BTS, EUPS and ESTI, whether products support the tasks required by each recommendation independent of guideline-based management integration, we calculated a task coverage score for each product–recommendation pair. For each nodule management recommendation, we summarized the specific tasks it requires in Supplementary Table S1. The task coverage score (0–100%) was defined as the proportion of these recommendation-defined tasks that were functionally supported by the product. Tasks within each recommendation were weighted equally to enable reproducible structural comparisons, and task coverage should be interpreted as functional overlap rather than clinical effectiveness or guideline concordance. Scores were visualized in heatmaps and grouped into four categories (low, moderate, substantial and high), with scoring rules detailed in Supplementary Table S2. To reflect that screening programs may deploy AI for different subsets of tasks (for example, detection and measurement only), we recalculated task coverage using alternative deployment scenarios, each with corresponding task weightings (Supplementary Table S3).

### Scientific evidence selection and analysis

Scientific evidence for each included AI product was identified using a two-step approach. First, we used the product–evidence dataset from Antonissen et al [17], which covered peer-reviewed publications on CE-certified radiological AI products between January 1, 2015, and March 31, 2023, and supplemented this by searching PubMed for additional articles published up to January 1, 2025, using product and vendor names (including former names, if applicable) as search terms (see Supplementary Table S4 for search queries). Second, vendor websites and the Health AI Register were manually reviewed to identify additional referenced publications. Studies were included if they evaluated the CE-marked version of the product (including earlier versions), reported performance on human datasets, were published in English in peer-reviewed journals, and assessed lung nodule analysis capabilities. Studies evaluating research-only or prototype software were excluded, as were reviews, protocols, commentaries, white papers, and case reports.

For each eligible study, data were extracted on study design (prospective or retrospective) and dataset characteristics, including use of lung cancer screening cohorts, multicenter or multi-country data collection, and scanner manufacturer diversity. Vendor independence was assessed through author affiliations, funding sources, and conflict of interest disclosures. To characterize evidence type, each study was categorized using the six-level hierarchical efficacy framework proposed by Antonissen et al and van Leeuwen et al [17, 18], adapted from Fryback and Thornbury [19]. This framework includes technical performance and diagnostic accuracy (levels 1–2), influence on clinical decision-making and patient outcomes (levels 3–5), and socioeconomic impact (level 6). Level definitions are provided in Table 1.

**Table 1:** Hierarchical model of efficacy to assess the contribution of AI software to the diagnostic imaging process, adapted from Fryback and Thornbury (1991)

Level	Explanation	Typical measures
Level 1t	**Technical efficacy** Article demonstrates the technical feasibility of the software.	Reproducibility, inter-software agreement, error rate.
Level 1c	**Potential clinical efficacy** Article demonstrates the feasibility of the software to be clinically applied.	Correlation to alternative methods, potential predictive value, biomarker studies.
Level 2	**Diagnostic accuracy efficacy** Article demonstrates the standalone performance of the software.	Standalone sensitivity, specificity, area under the ROC curve, or Dice score.
Level 3	**Diagnostic thinking efficacy** Article demonstrates the added value to the diagnosis.	Radiologist performance with/without AI, change in radiological judgment.
Level 4	**Therapeutic efficacy** Article demonstrates the impact of the software on the patient management decisions.	Effect on treatment or follow-up examinations.
Level 5	**Patient outcome efficacy** Article demonstrates the impact of the software on patient outcomes.	Effect on quality of life, morbidity or survival.
Level 6	**Societal efficacy** Article demonstrates the impact of the software on society by performing an economic analysis.	Effect on costs and quality-adjusted life years, incremental costs per quality-adjusted life year.

Level 1t: Level 1, technical; Level 1c: Level 1, clinical

## Results

### Recommendation-defined nodule management tasks and key differences

Comparison of the four nodule management recommendations showed substantial overlap in core lung cancer screening tasks, but also several task-specific differences (Supplementary Table S1). All define structured follow-up pathways for solid and subsolid nodules. Guidance for cystic and endobronchial nodules is provided only in Lung-RADS and ESTI. Measurement approaches differ, with BTS, EUPS, and ESTI favoring volumetric assessment, whereas Lung-RADS is primarily diameter-based, with optional volumetry. Malignancy risk assessment using the Pan-Canadian Early Detection of Lung Cancer (PanCan) model is integrated into BTS and EUPS, optional in Lung-RADS, and absent from ESTI.

### Overview of included AI products and their certified capabilities

**Fig. 1:**
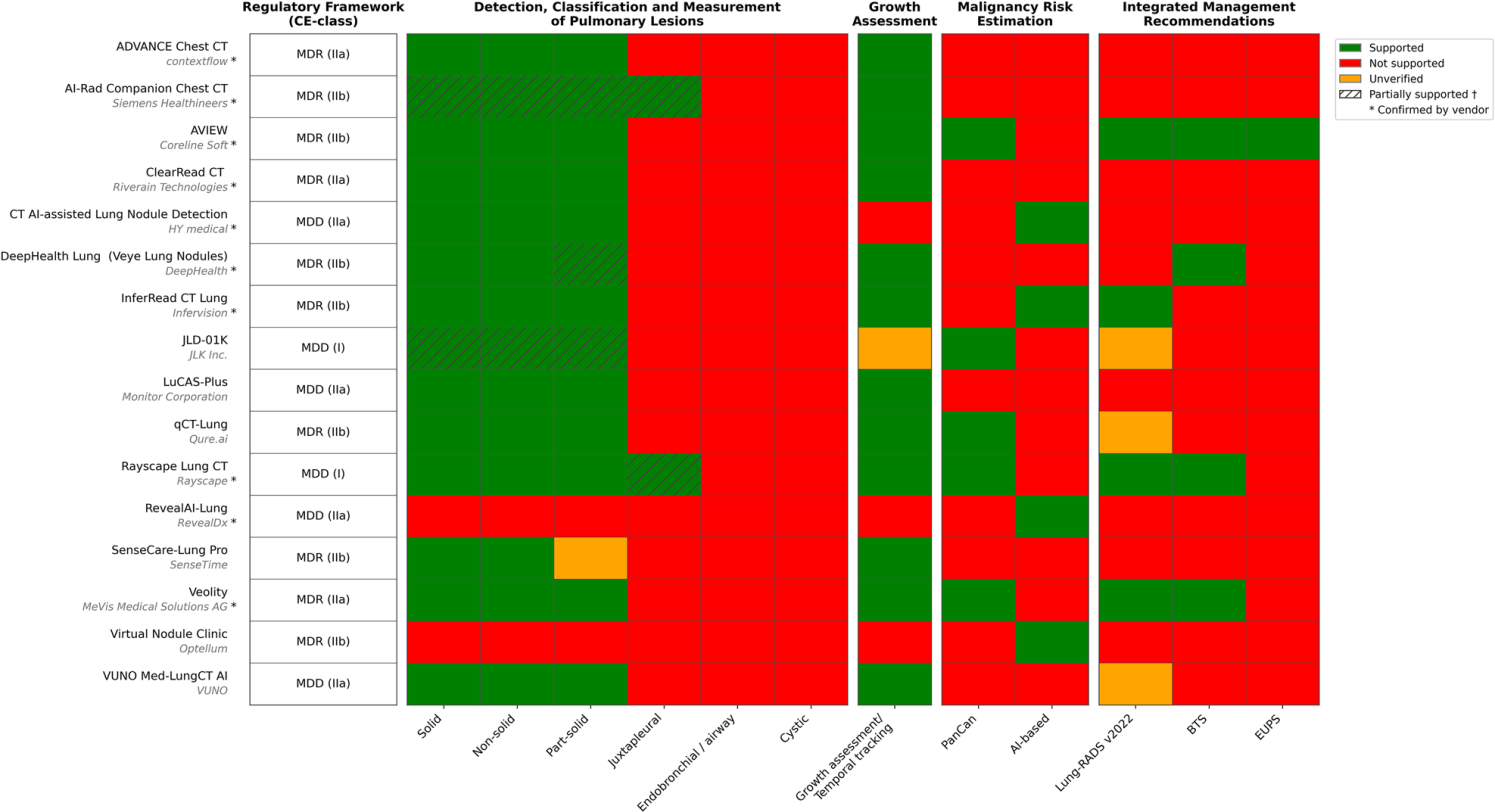
Capabilities of 16 CE-marked AI solutions for lung nodule analysis. The heatmap displays the presence or absence of capabilities across AI products based on public documentation and vendor responses. Green indicates supported functionality, red indicates not supported, and orange indicates unverified (suggested by public sources but documentation was incomplete or ambiguous and not confirmed by the vendor). Hatched cells indicate partial support (see footnote). Product and vendor names are shown on the left. Asterisks (*) denote vendor-confirmed information. † DeepHealth Lung (Veye Lung Nodules) supports detection for part-solid nodules but not classification or measurement; AI-Rad Companion Chest CT supports detection and measurement for solid, non-solid, part-solid, and juxtapleural nodules but not classification; Rayscape Lung CT supports detection and measurement for juxtapleural nodules but not classification; and JLD-01K supports detection and measurement for solid, non-solid, and part-solid nodules while classification remains unverified. BTS, British Thoracic Society; CE, Conformité Européenne; ESTI, European Society of Thoracic Imaging; EUPS, European Union Position Statement; Lung-RADS, Lung CT Screening Reporting and Data System; MDR, Medical Device Regulation; MDD, Medical Device Directive; PanCan, Pan-Canadian Early Detection of Lung Cancer

In total, 16 CE-marked AI products from 16 vendors for lung nodule analysis were identified. Of the 16 vendors, 10 (62.5%) completed our questionnaire; for the remaining 6, analysis relied on publicly available data. The regulatory certification status of these products is shown in Fig. 1, with 10 products (62.5%) certified under the Medical Device Regulation (7 class IIb, 3 class IIa) and 6 (37.5%) under the former, phased-out Medical Device Directive (4 class IIa, 2 class I).

As shown in Fig. 1, detection of solid and non-solid nodules is supported by 14/16 products each, and part-solid nodules by 13/16 (plus one unverified). In contrast, juxtapleural nodule detection is supported by two products, and none reported detection of endobronchial or cystic lesions. Classification is less common than detection, with 12 products classifying solid and non-solid nodules (plus one unverified each) and 10 classifying part-solid nodules (plus two unverified), while no products classify juxtapleural, endobronchial, or cystic lesions. Measurement is reported for solid and non-solid nodules in 14 products each and for part-solid nodules in 12 (plus one unverified), but is rare for juxtapleural nodules (*n* = 2) and absent for endobronchial and cystic lesions; diameter and volume measurements were not analyzed separately as all measurement-capable products report both.

Growth assessment was supported by 12 products (plus one unverified). Malignancy risk estimation was heterogeneous, with the PanCan model included in 5 products and AI-based risk scoring in 4. Guideline-linked management outputs were reported for Lung-RADS 2022 in 4 products, BTS in 4, and EUPS in 1, while an unspecified Lung-RADS version was reported in 3; 8 products reported no Lung-RADS, BTS, or EUPS management output.

### AI product task coverage across nodule management recommendations

**Fig. 2:**
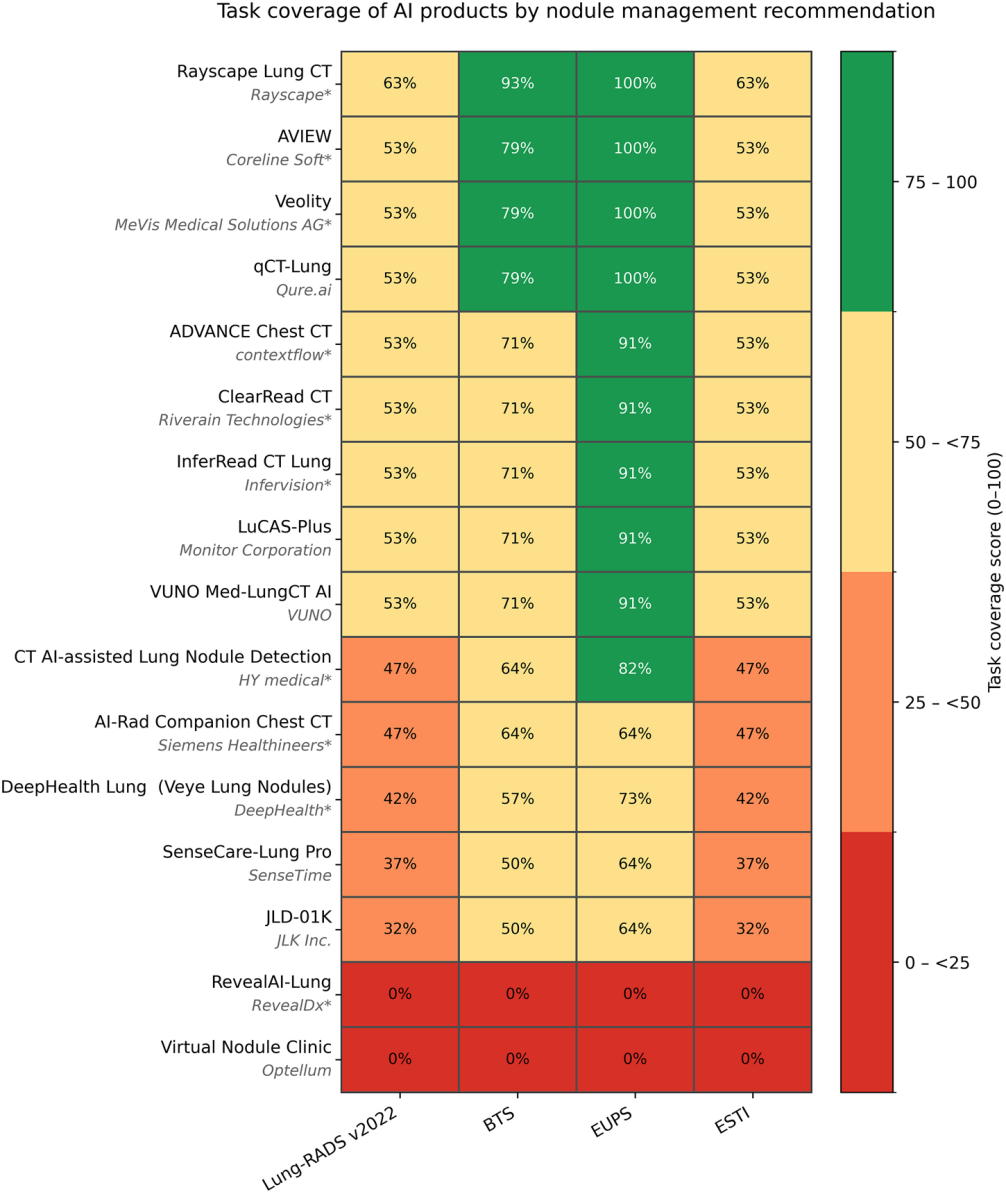
Task coverage of 16 CE-marked AI products across nodule management recommendations. Rows show vendor–product pairs; columns represent nodule management recommendations (Lung-RADS v2022, BTS, EUPS, and ESTI). Each cell shows the task coverage score (0–100), categorized as low (0 to < 25; red), moderate (25 to < 50; orange), substantial (50 to < 75; yellow), and high (75 to 100; green). Scores represent the equally weighted proportion of recommendation-defined tasks supported by each product, considering only tasks explicitly specified by each recommendation (tasks listed in Table S1; weighting and scoring rules in Table S2). Task coverage scores quantify functional overlap and do not measure performance, clinical effectiveness, or guideline concordance. Asterisks (*) indicate vendor-verified information. BTS, British Thoracic Society; ESTI, European Society of Thoracic Imaging; EUPS, European Union Position Statement; Lung-RADS, Lung CT Screening Reporting and Data System

Figure 2 shows, for each product, task coverage within each nodule management recommendation. Coverage was highest for EUPS, with 10/16 products achieving high coverage, 4/16 substantial coverage, and 2/16 low coverage. BTS showed the next highest coverage, with 4/16 products in the high category, 10/16 in the substantial category, and 2/16 in the low category. Lung-RADS v2022 and ESTI use the same task set and weights, resulting in identical scores and more modest coverage overall, with 9/16 products showing substantial coverage, 5/16 moderate coverage, and 2/16 low coverage.

In the scenario-based sensitivity analysis (Fig. S1), coverage was highest for screening-focused workflows limited to solid, part-solid and non-solid nodules: 13/16 products achieved high coverage in Scenario 1 (detection and measurement) and 11/16 in Scenarios 2 and 3 (adding classification and growth assessment, respectively). In contrast, when tasks were expanded to all lesion types (Scenario 4), no product achieved high coverage, and most showed only substantial coverage (9/16).

### Peer-reviewed evidence and study characteristics

**Fig. 3:**
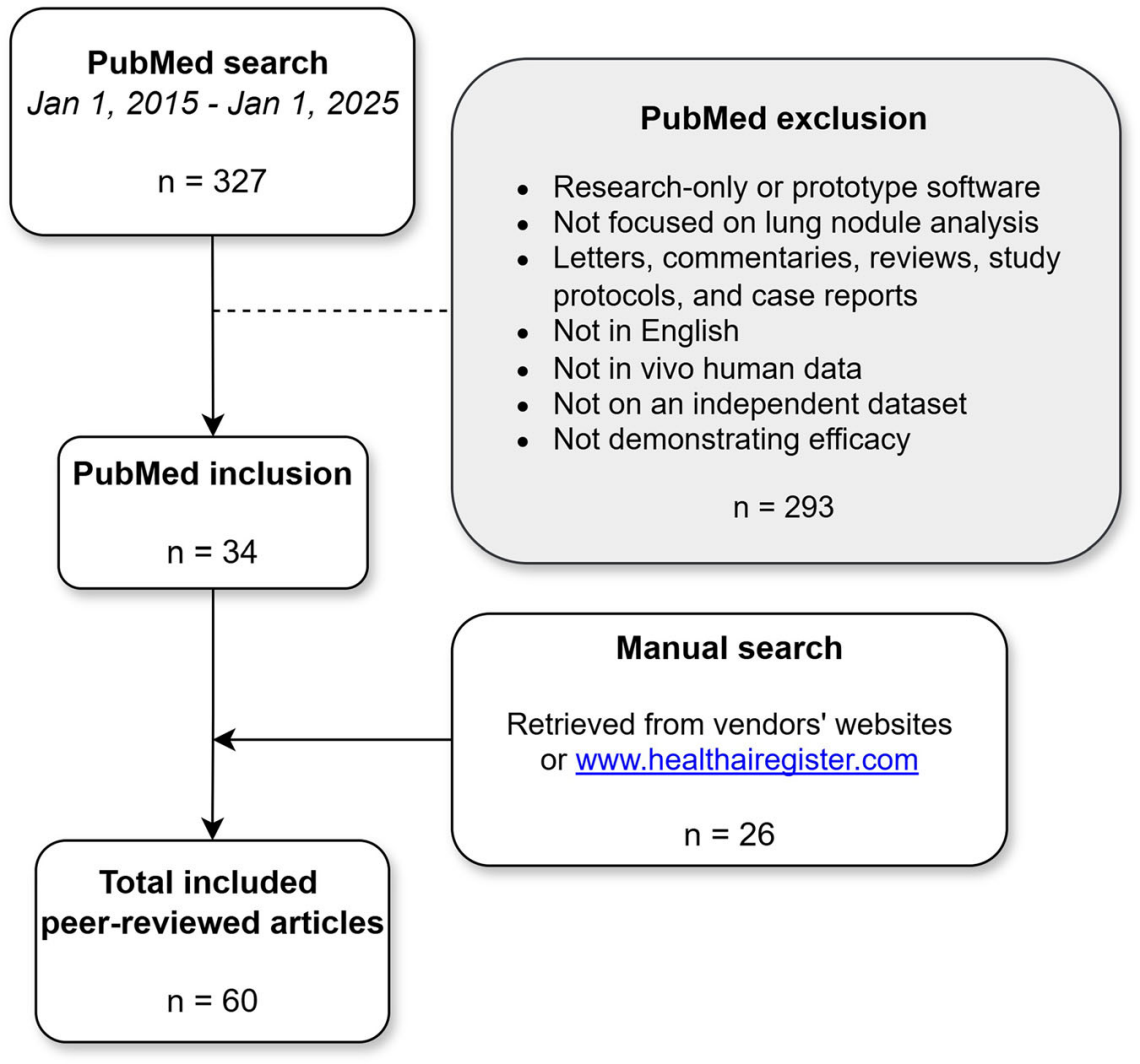
Flowchart summarizing the inclusion of peer-reviewed studies on CE-marked AI products for lung nodule analysis

The PubMed search identified 327 studies, of which 34 met the inclusion criteria. An additional 26 studies were included through manual searches, resulting in a total of 60 peer-reviewed studies included. Detailed search strategies and results are provided in Table S4, and the inclusion process is summarized in the flowchart shown in Fig. 3.

**Fig. 4:**
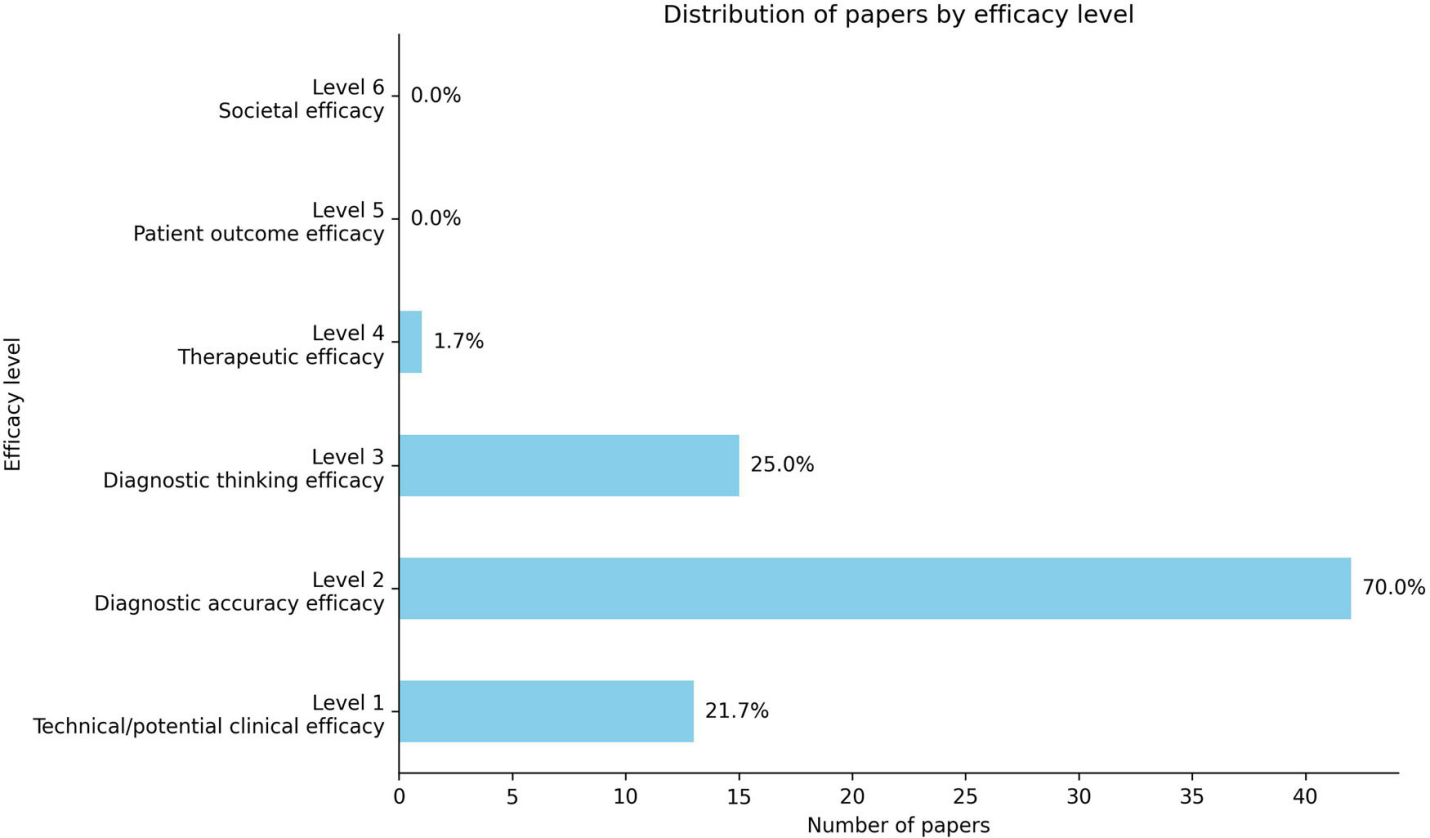
Levels of efficacy evaluated in the peer-reviewed papers included. The values next to each bar represent the percentage of papers at each efficacy level, relative to the total number of papers analyzed (*n* = 60). A single paper could address multiple levels of efficacy; therefore, the percentages add up to > 100%

Across the 60 peer-reviewed studies included, evidence was mainly concentrated at the lower levels of the hierarchical efficacy framework (Fig. 4). Diagnostic accuracy efficacy (Level 2) was assessed in 42 studies (70.0%), diagnostic thinking efficacy (Level 3) in 15 (25.0%), and technical or potential clinical efficacy (Level 1) in 13 (21.7%). Therapeutic efficacy (Level 4) was addressed in only 1 study (1.7%), and no study evaluated patient outcome efficacy (Level 5) or societal efficacy (Level 6).

**Fig. 5:**
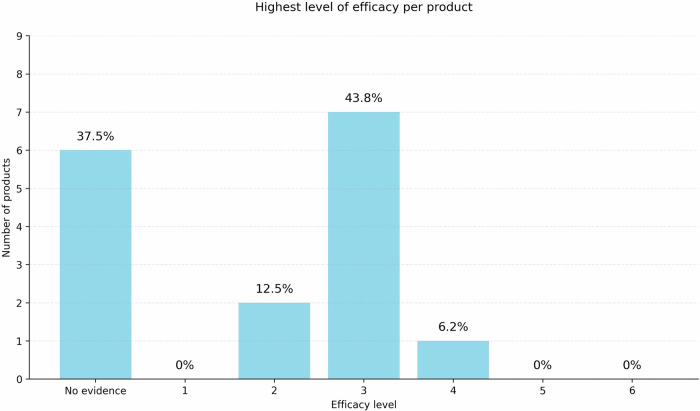
Highest validated efficacy level per included CE-marked AI product (*n* = 16) for chest CT lung nodule analysis

Of the 16 CE-marked AI products, 6 (37.5%) had no publicly available peer-reviewed evidence (Fig. 5). When considering the highest level of efficacy applicable for the remaining products, the highest validated level was Level 3 (diagnostic thinking efficacy) in 7/16 products (43.8%), Level 2 (diagnostic accuracy efficacy) in 2/16 (12.5%), and Level 4 (therapeutic efficacy) in 1/16 (6.2%). None of the products had their highest evidence at Level 1, Level 5, or Level 6.

The study design characteristics of included evidence are summarized in Table 2. Vendor-independent evaluations accounted for 27/60 studies (45.0%), and 4/60 studies (6.7%) assessed AI performance prospectively. External test datasets included lung cancer screening cohorts in 24/60 studies (40.0%) and routine clinical populations in 42/60 studies (70.0%). Across these datasets, 24/60 studies (40.0%) used multicenter data, 4/60 (6.7%) used multinational data, and 15/60 (25.0%) included scans acquired on devices from multiple CT manufacturers.

**Table 2:** Summary of research study characteristics, including total count, vendor independence, prospective assessment, and external test data characteristics

Metric	Count
Total studies	60 (100%)
Independent of the vendor	27 (45.0%)
AI performance assessed in a prospective setting	4 (6.7%)
External test data:	
Lung cancer screening data	24 (40.0%)
Clinical routine care data	42 (70.0%)
Multicenter	24 (40.0%)
Multinational	4 (6.7%)
Acquired from multiple manufacturers	15 (25.0%)

An overview of all included studies with their respective efficacy level, study design characteristics, and assessed capabilities is provided in the supplemental materials (Appendix E2).

## Discussion

This study provides a structured overview of 16 CE-marked AI products for lung nodule analysis in lung cancer screening. We mapped product capabilities, quantified their functional coverage of tasks defined by four international nodule management recommendations (Lung CT Screening Reporting and Data System (Lung-RADS) v2022 [13], British Thoracic Society (BTS) pulmonary nodule guidelines [14], European Union Position Statement (EUPS) recommendations [15], and European Society of Thoracic Imaging (ESTI) recommendations [16]), and assessed the peer-reviewed evidence available for each product.

Most products support the core screening workflow for solid and subsolid nodules, including detection, volumetric measurement, and growth assessment, and several additionally provide malignancy risk estimation using the Pan-Canadian Early Detection of Lung Cancer (PanCan) model [20]. These capabilities overlap strongly with the EUPS and BTS task sets, explaining the higher task coverage observed for these recommendations. However, none of the evaluated products provides CE-marked support for endobronchial or cystic lesions, which are explicitly addressed in Lung-RADS and ESTI. Consequently, current commercial tools can assist with key screening tasks, but they do not yet meet the full requirements of Lung-RADS- or ESTI-based pathways. The scenario-based analysis reinforces this pattern: task coverage is high for common nodule workflows, but drops sharply once the broader lesion spectrum is included.

These capability gaps highlight practical considerations for implementing AI in lung cancer screening. Product choice should be aligned with the intended nodule management recommendations to be followed, and it is useful to map each product’s capabilities against key nodule management requirements to identify residual gaps. Combining multiple AI products to cover all required functionality is possible but typically undesirable because it increases workflow and integration complexity [21]. For developers, these gaps can inform future development priorities by highlighting recommendation-defined tasks that remain unsupported across the market. Regulatory status is an additional consideration, as several products were still certified under the former Medical Device Directive and will have to transition to the Medical Device Regulation [22] by December 2028 to stay on the market. This may affect product continuity, upgrade paths, and long-term procurement planning.

Of the 16 products, 6 had no peer-reviewed evidence, while the remaining 10 were supported by 60 studies that were predominantly retrospective, included lung cancer screening cohorts in only 40%, and focused mainly on lower-efficacy outcomes such as technical performance and diagnostic accuracy. Higher-efficacy evidence was less common: only a quarter examined effects on clinical decision-making, and none assessed patient outcomes or societal impact. The limited availability of higher-efficacy evidence may in part reflect that organized LDCT lung cancer screening is still variably implemented across Europe, with many settings still in pilot or early rollout stages [5], which limits opportunities to generate prospective, program-level evidence with longer follow-up. Overall, the literature supports technical validation and comparative assessment of products but provides a limited basis to determine real-world impact in screening practice.

Nevertheless, the available evidence provides useful information on how AI may affect screening performance. Geppert et al [23] systematically reviewed test-accuracy studies of CE-marked AI in CT lung cancer screening and found that AI assistance tended to increase sensitivity, often at the cost of lower specificity and more false-positive findings. These trade-offs support introducing AI with predefined performance thresholds and ongoing quality assurance, including monitoring key metrics such as recall rates and invasive procedures [10]. Breast cancer screening offers a strong template for how this can be implemented at scale: a larger and more mature evidence base, including prospective trials such as the MASAI trial [24], has shaped how AI is compared with local standards, evaluated alongside human readers, and integrated into structured quality assurance systems. Lung cancer screening programs could adopt similar requirements for prospective evaluation in true screening cohorts before AI is given a central role.

A further implementation consideration is how to use AI-based malignancy risk estimation. Validated clinical risk models such as PanCan [20] are incorporated in BTS and EUPS recommendations, whereas AI-derived risk scores are not yet integrated into management guidance and are best regarded as adjunctive decision support. Although AI-based risk estimation shows promising performance in research settings [25, 26], recommendations do not define how to act on AI-based risk thresholds or link them to management pathways. Nonetheless, the growing availability of risk estimation in commercial tools suggests a potential future shift from morphology-based nodule management toward more integrated risk stratification if consensus thresholds and guideline-linked pathways are established.

Our study has several limitations. First, capability mapping relied on vendor-reported data and publicly available documentation because detailed functionality information for CE-marked AI products is not publicly accessible, limiting independent verification. For non-responders, capabilities were derived from public sources that may be incomplete or commercially framed and should not be interpreted as independently verified clinical functionality. The European Commission is working on a public medical device register called Eudamed; however, this register is not yet filled and lacks details on  product functionality and validation activities. Greater transparency in regulatory documentation, such as the publicly accessible database maintained by the U.S. Food and Drug Administration, would improve independent evaluation of AI products. Second, task coverage scores quantify functional overlap and should not be interpreted as measures of clinical effectiveness or guideline concordance. We used equal weighting to enable a reproducible comparison, although the clinical importance of individual tasks is not equal in practice. To address this, we performed sensitivity analyses using alternative weighting scenarios, which did not change the main conclusions of the study. Finally, the analysis focused on nodule management and excluded incidental findings such as emphysema and coronary calcification scoring, since these were considered beyond the scope of this work.

In conclusion, CE-marked AI products for lung cancer screening now provide support for a substantial share of tasks needed to follow current nodule management recommendations, but important capability gaps and a limited prospective evidence base remain. Our overview offers a structured basis for capability-based procurement and local evaluation planning, yet adoption should remain conditional on program-specific validation, workflow integration, and ongoing quality assurance. Future research should prioritize prospective post-deployment studies in real screening programs that report clinical and program outcomes, workflow impact, generalizability, equity, and governance. As lung cancer screening programs expand, generating and publishing such real-world evidence will be crucial for responsible AI adoption at scale.

## Supplementary information

ELECTRONIC SUPPLEMENTARY MATERIAL

Appendix_E2_clean.

## Abbreviations

AI: Artificial intelligence
BTS: British Thoracic Society
CE: Conformité Européenne/European Conformity
ESTI: European Society of Thoracic Imaging
EUPS: European Union Position Statement
LCS: Lung cancer screening
LDCT: Low-dose computed tomography
Lung-RADS: Lung CT Screening Reporting and Data System
PanCan: Pan-Canadian Early Detection of Lung Cancer

## References

[1] Bray F, Laversanne M, Sung H et al (2024) Global cancer statistics 2022: GLOBOCAN estimates of incidence and mortality worldwide for 36 cancers in 185 countries. CA Cancer J Clin 74:229–263. 10.3322/caac.21834 38572751 10.3322/caac.21834

[2] National Lung Screening Trial Research Team (2011) Reduced lung-cancer mortality with low-dose computed tomographic screening. N Engl J Med 365:395–409. 10.1056/NEJMoa1102873 21714641 10.1056/NEJMoa1102873 PMC4356534

[3] De Koning HJ, Van Der Aalst CM, De Jong PA et al (2020) Reduced lung-cancer mortality with volume CT screening in a randomized trial. N Engl J Med 382:503–513. 10.1056/NEJMoa1911793 31995683 10.1056/NEJMoa1911793

[4] European Commission, Directorate-General for Research and Innovation, Group of Chief Scientific Advisors (2022) Cancer screening in the European Union. Publications Office of the European Union. Available via https://data.europa.eu/doi/10.2777/867180. Accessed 25 June 2025

[5] Lung Cancer Policy Network (2025) Interactive map of lung cancer screening (third edition). Available via https://www.lungcancerpolicynetwork.com/interactive-map-of-lung-cancer-screening. Accessed 1 Sept 2025

[6] Wait S, Alvarez-Rosete A, Osama T et al (2022) Implementing lung cancer screening in Europe: taking a systems approach. JTO Clin Res Rep 3:100329. 10.1016/j.jtocrr.2022.100329 35601926 10.1016/j.jtocrr.2022.100329 PMC9121320

[7] Gleeson F, Revel MP, Biederer J et al (2023) Implementation of artificial intelligence in thoracic imaging—a what, how, and why guide from the European Society of Thoracic Imaging (ESTI). Eur Radiol 33:5077–5086. 10.1007/s00330-023-09409-2 36729173 10.1007/s00330-023-09409-2 PMC9892666

[8] Chassagnon G, De Margerie-Mellon C, Vakalopoulou M et al (2023) Artificial intelligence in lung cancer: current applications and perspectives. Jpn J Radiol 41:235–244. 10.1007/s11604-022-01359-x 36350524 10.1007/s11604-022-01359-x PMC9643917

[9] Radiology Health AI Register (2025). Available via https://www.healthairegister.com/radiology/products. Accessed 25 June 2025

[10] Hahn HK, May MS, Dicken V et al (2025) Requirements for quality assurance of AI models for early detection of lung cancer. Preprint at https://arxiv.org/abs/2502.17639

[11] NICE (2023) Committee discussion. AI-derived computer-aided detection (CAD) software for detecting and measuring lung nodules in CT scan images. Guidance. Available via https://www.nice.org.uk/guidance/dg55/chapter/3-Committee-discussion. Accessed 22 Sept 2025

[12] Federal Ministry for the Environment, Nature Conservation, Nuclear Safety and Consumer Protection (2024) Ordinance on the Permissibility of Using Low-Dose Computed Tomography for the Lung Cancer Screening of Smokers in the version of 15 May 2024 (Federal Law Gazette 2024 I No. 162). Available via https://www.gesetze-im-internet.de/englisch_lukrfr_herkv/englisch_lukrfr_herkv.html#p0051. Accessed 22 Sept 2025

[13] American College of Radiology Committee on Lung-RADS® (2022) Lung-RADS assessment categories 2022. Available via https://www.acr.org/Clinical-Resources/Clinical-Tools-and-Reference/Reporting-and-Data-Systems/Lung-RADS. Accessed 25 May 2025

[14] Callister MEJ, Baldwin DR, Akram AR et al (2015) British Thoracic Society guidelines for the investigation and management of pulmonary nodules: accredited by NICE. Thorax 70:ii1–ii54. 10.1136/thoraxjnl-2015-207168 26082159 10.1136/thoraxjnl-2015-207168

[15] Oudkerk M, Devaraj A, Vliegenthart R et al (2017) European position statement on lung cancer screening. Lancet Oncol 18:e754–e766. 10.1016/S1470-2045(17)30861-6 29208441 10.1016/S1470-2045(17)30861-6

[16] Prokop M, Schaefer-Prokop C, Jacobs C et al (2025) Aggressiveness-guided nodule management for lung cancer screening in Europe—justification for follow-up intervals and definition of growth. Eur Radiol. 10.1007/s00330-025-11647-5 10.1007/s00330-025-11647-5 PMC12712097 40593169

[17] Antonissen N, Tryfonos O, Houben IB, Jacobs C, De Rooij M, Van Leeuwen KG (2025) Artificial intelligence in radiology: 173 commercially available products and their scientific evidence. Eur Radiol. 10.1007/s00330-025-11830-8 10.1007/s00330-025-11830-8 PMC12711992 40707732

[18] Van Leeuwen KG, Schalekamp S, Rutten MJCM, Van Ginneken B, De Rooij M (2021) Artificial intelligence in radiology: 100 commercially available products and their scientific evidence. Eur Radiol 31:3797–3804. 10.1007/s00330-021-07892-z 33856519 10.1007/s00330-021-07892-z PMC8128724

[19] Fryback DG, Thornbury JR (1991) The efficacy of diagnostic imaging. Med Decis Making 11:88–94. 10.1177/0272989X9101100203 1907710 10.1177/0272989X9101100203

[20] McWilliams A, Tammemagi MC, Mayo JR et al (2013) Probability of cancer in pulmonary nodules detected on first screening CT. N Engl J Med 369:910–919. 10.1056/NEJMoa1214726 24004118 10.1056/NEJMoa1214726 PMC3951177

[21] Korfiatis P, Kline TL, Meyer HM et al (2025) Implementing artificial intelligence algorithms in the radiology workflow: challenges and considerations. Mayo Clin Proc Digit Health 3:100188. 10.1016/j.mcpdig.2024.100188 40207002 10.1016/j.mcpdig.2024.100188 PMC11975811

[22] EUR-Lex (2025) Regulation (EU) 2017/745 of the European Parliament and of the Council of 5 April 2017 on Medical Devices, Amending Directive 2001/83/EC, Regulation (EC) No 178/2002 and Regulation (EC) No 1223/2009 and Repealing Council Directives 90/385/EEC and 93/42/EEC (Text with EEA Relevance). Available via http://data.europa.eu/eli/reg/2017/745/2025-01-10/eng. Accessed 29 Sept 2025

[23] Geppert J, Asgharzadeh A, Brown A et al (2024) Software using artificial intelligence for nodule and cancer detection in CT lung cancer screening: systematic review of test accuracy studies. Thorax 79:1040–1049. 10.1136/thorax-2024-221662 39322406 10.1136/thorax-2024-221662 PMC11503082

[24] Hernström V, Josefsson V, Sartor H et al (2025) Screening performance and characteristics of breast cancer detected in the Mammography Screening with Artificial Intelligence trial (MASAI): a randomised, controlled, parallel-group, non-inferiority, single-blinded, screening accuracy study. Lancet Digit Health 7:e175–e183. 10.1016/S2589-7500(24)00267 39904652 10.1016/S2589-7500(24)00267-X

[25] Baldwin DR, Gustafson J, Pickup L et al (2020) External validation of a convolutional neural network artificial intelligence tool to predict malignancy in pulmonary nodules. Thorax 75:306–312. 10.1136/thoraxjnl-2019-214104 32139611 10.1136/thoraxjnl-2019-214104 PMC7231457

[26] Adams SJ, Madtes DK, Burbridge B et al (2023) Clinical impact and generalizability of a computer-assisted diagnostic tool to risk-stratify lung nodules with CT. J Am Coll Radiol 20:232–242. 10.1016/j.jacr.2022.08.006 36064040 10.1016/j.jacr.2022.08.006

