# Supplementary material for: Commercial AI for CT lung cancer screening: product capabilities, coverage of nodule management tasks and supporting evidence
Source: Eur Radiol. 2026 May 7;36(9):7199–209. doi: 10.1007/s00330-026-12580-x (PMC13451236; doi:10.1007/s00330-026-12580-x)
Supplement: Supplementary file 1 [file 330_2026_12580_MOESM1_ESM.pdf]

Commercial AI for CT lung cancer screening: product capabilities, coverage of nodule management tasks and supporting evidence

ELECTRONIC SUPPLEMENTARY MATERIAL

Appendix E1. Questionnaire sent to vendors. The questionnaire was distributed by email as a link to a Google Form for vendors to confirm or update the applicable capabilities of their CE-certified product. For transparency, the complete list of questions was also provided in PDF format, allowing vendors to preview all items before completing the form.

Review of CE-certified AI functionalities for lung cancer screening

This document contains all the questions included in the online form and is provided to help you prepare your responses. The questions focus on the automatic, CE-certified functionalities of your AI solution relevant to low-dose CT lung cancer screening.

Please note: This PDF is for preview purposes only. The official submission must be completed via the personalized Google Form link provided in the email.

General product information:

1. Please provide the current product name and the name of the company that holds the CE certification.
2. Which version of your product is currently CE-certified and forms the basis for the functionality details provided below?
3. Under which CE certification framework is your current product approved, and what is its classification?
    - MDR – Class I
    - MDR – Class IIa
    - MDR – Class IIb
    - MDR – Class III
    - MDD – Class I
    - MDD – Class IIa
    - MDD – Class IIb
    - MDD – Class III

Nodule detection:

4. Does your CE-certified product automatically detect pulmonary nodules?
   - Yes
   - No
5. Which types of pulmonary lesions can your CE-certified product automatically detect?
   - Solid nodules
   - Non-solid nodules
   - Part-solid nodules
   - Juxtapleural nodules
   - Endobronchial/airway lesions
   - (Atypical) cystic lesions
   - The product can automatically detect nodules, but specific lesion types are not specified in available documentation
   - Other

Nodule classification:

6. Does your CE-certified product automatically classify pulmonary nodules based on their type or characteristics (e.g., solid, non-solid, juxtapleural)?
   - Yes
   - No
7. Which types of pulmonary lesions can your CE-certified product automatically classify by type or characteristics?
   - Solid nodules
   - Non-solid nodules
   - Part-solid nodules
   - Juxtapleural nodules
   - Endobronchial/airway lesions
   - (Atypical) cystic lesions
   - Other

Pulmonary nodule measurement and quantification:

8. Does your CE-certified product automatically measure pulmonary nodules by volume and/or diameter?
   - Yes
   - No

9. Please specify which measurements are provided <u>automatically</u>:
    - Volume (e.g., mm³)
    - Diameter (e.g., mm)
    - Other
10. For which types of pulmonary lesions does your CE-certified product <u>automatically</u> provide measurements (e.g., volume and/or diameter)?
    - Solid nodules
    - Non-solid nodules
    - Part-solid nodules
    - Juxtapleural nodules
    - Endobronchial/airway lesions
    - (Atypical) cystic lesions
    - The product can automatically measure nodules, but specific lesion types are not specified in available documentation
    - Other
11. Does the product <u>automatically</u> compare pulmonary nodule measurements (e.g., volume and/or diameter) across multiple time points?
    - Yes
    - No
12. Which pulmonary nodule growth metrics does the product <u>automatically</u> provide?
    - Volume doubling time (VDT)
    - Growth in volume (absolute or % change over time)
    - Growth in diameter (absolute or % change over time)
    - Other

Malignancy risk estimation:

13. Does your CE-certified product provide malignancy risk estimation at the nodule and/or participant level?
    - Yes
    - No
14. At what level does your CE-certified product estimate malignancy risk?
    - Per nodule (individual lesion-level risk)
    - Per participant (overall participant-level risk)
    - Other

15. Which model(s) are used for malignancy risk estimation in your CE-certified product?
    - AI-based malignancy risk scoring
    - Brock/PanCan Model
    - Mayo Clinic Model
    - Other

Structured reporting and management recommendations:

16. Does your product include structured reporting with embedded management recommendations (e.g., Lung-RADS, BTS guidelines)?
    - Yes
    - No
17. Which guideline(s) or management recommendation frameworks are integrated into your CE-certified product's structured reporting?
    - Lung-RADS version 1.0 or 1.1 (2019) - (https://www.acr.org/Clinical-Resources/Clinical-Tools-and-Reference/Reporting-and-Data-Systems/Lung-RADS)
    - Lung-RADS version 2.0 (2022) - (https://www.acr.org/Clinical-Resources/Clinical-Tools-and-Reference/Reporting-and-Data-Systems/Lung-RADS)
    - BTS (British Thoracic Society) - (https://www.brit-thoracic.org.uk/quality-improvement/guidelines/pulmonary-nodules/)
    - European Union Position Statement on Lung Cancer Screening (Oudkerk M et al. European position statement on lung cancer screening. Lancet Oncol. 2017;18(12):e754-e766. doi:10.1016/S1470-2045(17)30861-6)/
    - Fleischner 2017 guideline (MacMahon H al. Guidelines for Management of Incidental Pulmonary Nodules Detected on CT Images: From the Fleischner Society 2017. Radiology. 2017;284(1):228-243. doi:10.1148/radiol.2017161659)
    - Other

Additional information:

18. Is your CE-marked AI product currently being used in any lung cancer screening settings, such as national screening programs or clinical trials? If so, please indicate the countries and specify the names of the trials or national programs where applicable
19. Do you have any additional information, remarks, or recent updates you would like to share about your CE-certified product?

Table S1. Summary of standardized clinical tasks across four major nodule management recommendations (Lung-RADS version 2022, BTS, EUPS, ESTI), grouped into functional domains relevant to lung nodule analysis.

Task domain	Subtask	Lung-RADS v2022	BTS	EUPS	ESTI	Notes
**Detection, Classification & Measurement**	Solid nodules	✓	✓	✓	✓	All address solid nodules.
	Subsolid nodules (part-solid, non-solid)	✓	✓	✓	✓	All address subsolid nodules.
	Juxtapleural nodules	✓	✓	x	✓	Lung-RADS, BTS, and ESTI label typical intrapulmonary lymph node (ILN) morphology as benign (solid, smooth, oval/lentiform/triangular, <10 mm, and ≤1 cm from the pleura). BTS and ESTI do not explicitly use the term “juxtapleural,” but these lesions fall within their ILN criteria. EUPS acknowledges ILN morphology and cautions against size-only management but provides no specific pathway.
	(Atypical) cystic lesions	✓	x	x	✓	Both Lung-RADS and ESTI provide dedicated pathways for (atypical) cystic lesions. BTS acknowledges cavity wall thickness as a malignancy predictor but does not provide a cyst-specific management pathway. EUPS does not address cystic lesions separately.
	Endobronchial / airway lesions	✓	x	x	✓	Both Lung-RADS and ESTI provide dedicated pathways for airway (endobronchial) nodules. BTS does not include airway lesions in its pulmonary nodule screening protocol. EUPS does not address endobronchial/airway lesions separately.
**Measurement & Growth Assessment**	Measurement — Diameter	✓	~	~	~	Lung-RADS uses diameter measurements as the primary method for nodule classification, whereas BTS, EUPS, and ESTI recommend volumetry as the preferred method, applying diameter thresholds only when volumetry is not feasible.
	Measurement — Volumetry	~	✓	✓	✓	Volumetry is optional in Lung-RADS ('if obtained'); standard in BTS, EUPS, ESTI.

	Growth — Diameter change	✓	~	~	~	Lung-RADS defines growth as an increase in mean diameter of >1.5 mm within 12 months. In contrast, BTS, EUPS, and ESTI rely primarily on volumetry, using diameter measurements only as a fallback when volumetric assessment is not feasible
	Growth — Volume change (VDT)	x	✓	✓	✓	Lung-RADS does not calculate VDT; BTS/EUPS/ESTI use defined VDT cut-offs to guide work-up. ESTI adjusts VDT thresholds to scan intervals and limits allowable absolute diameter growth (≤5 mm).
**Malignancy Risk Estimation**	Integration of malignancy risk models (e.g., PanCan model)	~	✓	✓	x	BTS integrates the PanCan (Brock) model within its management pathway, using calculated risk to guide downstream actions. EUPS adopts the BTS subsolid pathway, including malignancy risk estimation by the PanCan model. Lung-RADS allows PanCan as an optional adjunct (not category-defining), and ESTI does not incorporate a validated malignancy risk model.
**Structured Management**	Defined follow-up and decision pathways	✓	✓	✓	✓	All provide structured management frameworks: BTS, EUPS, and ESTI provide flowchart-style management pathways, whereas Lung-RADS applies a category-based system, all having predefined follow-up intervals.

Symbols denote whether a task is explicitly defined (✓), partially addressed (~), or not addressed (x).

BTS, British Thoracic Society; *ESTI*, European Society of Thoracic Imaging; *EUPS*, European Union Position Statement; *ILN,* intrapulmonary lymph node; *Lung-RADS*, Lung CT Screening Reporting and Data System; *VDT*, volume doubling time; *PanCan,* Pan-Canadian Early Detection of Lung Cancer

Table S2. Task weight matrix by nodule management recommendation

Task domain	Subtask	Lung-RADS v2022	BTS	EUPS	ESTI
**Detection**	Solid nodules	5.263158	7.142857	9.090909	5.263158
	Non-solid nodules	5.263158	7.142857	9.090909	5.263158
	Part-solid nodules	5.263158	7.142857	9.090909	5.263158
	Juxtapleural nodules	5.263158	7.142857	0	5.263158
	(Atypical) cystic lesions	5.263158	0	0	5.263158
	Endobronchial / airway lesions	5.263158	0	0	5.263158
**Classification**	Solid nodules	5.263158	7.142857	9.090909	5.263158
	Non-solid nodules	5.263158	7.142857	9.090909	5.263158
	Part-solid nodules	5.263158	7.142857	9.090909	5.263158
	Juxtapleural nodules	5.263158	7.142857	0	5.263158
	(Atypical) cystic lesions	5.263158	0	0	5.263158
	Endobronchial / airway lesions	5.263158	0	0	5.263158
**Measurement***	Solid nodules	5.263158	7.142857	9.090909	5.263158
	Non-solid nodules	5.263158	7.142857	9.090909	5.263158
	Part-solid nodules	5.263158	7.142857	9.090909	5.263158
	Juxtapleural nodules	5.263158	7.142857	0	5.263158
	(Atypical) cystic lesions	5.263158	0	0	5.263158
	Endobronchial / airway lesions	5.263158	0	0	5.263158
**Growth assessment***		5.263158	7.142857	9.090909	5.263158
**Malignancy risk estimation using the PanCan-model**		0	7.142857	9.090909	0

Tasks were derived from *Table S1*, and for each of the four nodule management recommendations only explicitly specified tasks were assigned a weight, while tasks that were partially addressed or not addressed were given a weight of 0. Within each recommendation, explicit items were weighted equally and renormalized to sum to 100% (effective per-item weight = 100 / number of explicit items).

*No distinction was made between diameter- and volume-based measurement or growth assessment because all products reporting these features provided both diameter and volume outputs and volume-doubling time (VDT).

BTS, British Thoracic Society; *ESTI*, European Society of Thoracic Imaging; *EUPS*, European Union Position Statement; *Lung-RADS*, Lung CT Screening Reporting and Data System; *PanCan,* Pan-Canadian Early Detection of Lung Cancer

Table S3. Task weight matrix for the scenario-based sensitivity analysis

Task domain	Subtask	Scenario 1	Scenario 2	Scenario 3	Scenario 4
**Detection**	Solid nodules	16.666667	11.111111	10	5.263158
	Non-solid nodules	16.666667	11.111111	10	5.263158
	Part-solid nodules	16.666667	11.111111	10	5.263158
	Juxtapleural nodules	0	0	0	5.263158
	(Atypical) cystic lesions	0	0	0	5.263158
	Endobronchial / airway lesions	0	0	0	5.263158
**Classification**	Solid nodules	0	11.111111	10	5.263158
	Non-solid nodules	0	11.111111	10	5.263158
	Part-solid nodules	0	11.111111	10	5.263158
	Juxtapleural nodules	0	0	0	5.263158
	(Atypical) cystic lesions	0	0	0	5.263158
	Endobronchial / airway lesions	0	0	0	5.263158
**Measurement***	Solid nodules	16.666667	11.111111	10	5.263158
	Non-solid nodules	16.666667	11.111111	10	5.263158
	Part-solid nodules	16.666667	11.111111	10	5.263158
	Juxtapleural nodules	0	0	0	5.263158
	(Atypical) cystic lesions	0	0	0	5.263158
	Endobronchial / airway lesions	0	0	0	5.263158
**Growth assessment***		0	0	10	5.263158
**Malignancy risk estimation using the PanCan-model**		0	0	0	0

We performed a scenario-based sensitivity analysis to reflect that, in routine lung cancer screening, AI products are not necessarily deployed to reproduce full guideline pathways end-to-end. Instead, programs may use AI to support selected workflow components depending on local protocols and the intended role. The scenarios vary along two dimensions: the scope of lesion types included and whether longitudinal assessment is required when prior examinations are available. ***Scenario 1*** includes detection and measurement for solid, non-solid, and part-

solid nodules only. ***Scenario 2*** adds classification of these nodule types to support pathway selection and reporting consistency. ***Scenario 3*** further adds growth assessment for these nodule types to reflect subsequent screening rounds or follow-up where longitudinal comparison drives management. ***Scenario 4*** broadens the lesion-type scope by including detection, measurement, and classification across all lesion categories while retaining growth assessment, representing a more comprehensive longitudinal workflow. Within each scenario, included tasks were weighted equally, and weights were renormalized to sum to 100%, consistent with the approach used for the main task-weight matrices in *Table S2*. Malignancy risk estimation was assigned a weight of zero in all scenarios.

*No distinction was made between diameter- and volume-based measurement or growth assessment because all products reporting these features provided both diameter and volume outputs and volume-doubling time (VDT).

PanCan, Pan-Canadian Early Detection of Lung Cancer

Table S4. Literature search queries, hits, and included studies per vendor.

Vendor	Query PubMed	Hits PubMed	Included PubMed	Included manual	Included total
**DeepHealth**	(("2015"[Date - Publication] : "3000"[Date - Publication])) AND (Aidence OR Deephealth OR "veye chest" OR "veye lung nodules" OR "saige lung")	74	3	2	5
**Contextflow**	(("2015"[Date - Publication] : "3000"[Date - Publication])) AND (Contextflow) AND (''chest CT'')	12	0	0	0
**Coreline Soft**	(("2015"[Date - Publication] : "3000"[Date - Publication])) AND (''Coreline Soft'') AND (''chest CT'' OR ''LungScreen'' OR ''AVIEW LCS'' OR ''AVIEW-LCS'')	26	5	4	9
**Infervision**	(("2015"[Date - Publication] : "3000"[Date - Publication])) AND (Infervision OR Inferread) AND (''chest CT'')	32	5	2	7
**JLK Inc.**	(("2015"[Date - Publication] : "3000"[Date - Publication])) AND (JLK) AND (JLD-01K OR ''chest CT'')	4	0	0	0
**MeVis Medical Solutions AG**	(("2015"[Date - Publication] : "3000"[Date - Publication])) AND (''MeVis Medical Solutions'') AND (Veolity OR ''chest CT'')	26	2	10	12
**Monitor Corporation**	(("2015"[Date - Publication] : "3000"[Date - Publication])) AND (''Monitor Corporation'') AND (LuCAS-Plus OR ''chest CT'')	16	0	0	0
**Optellum**	(("2015"[Date - Publication] : "3000"[Date - Publication])) AND (Optellum) AND (''Nodule Clinic'' OR ''chest CT'')	32	7	2	9
**Qure.ai**	(("2015"[Date - Publication] : "3000"[Date - Publication])) AND (Qure.ai) AND (''qCT-lung'' OR ''chest CT'')	10	1	0	1
**Rayscape**	(("2015"[Date - Publication] : "3000"[Date - Publication])) AND (Rayscape) AND (Xvision OR CTXVISION OR ''chest CT'')	1	0	0	0
**RevealDx**	(("2015"[Date - Publication] : "3000"[Date - Publication])) AND ((RevealDx OR "Mindshare Medical" AND (''RevealAI-Lung'' OR ''chest CT''))	2	1	0	1
**Riverain Technologies**	("2015"[Date - Publication] : "3000"[Date - Publication])) AND (Riverain AND ''chest CT) OR (''ClearRead CT'') OR (''ClearReadCT'')	18	6	3	9
**SenseTime**	(("2015"[Date - Publication] : "3000"[Date - Publication])) AND (SenseTime) AND (''SenseCare'' OR ''SenseCare-Lung Pro'' OR ''chest CT'')	1	0	0	0
**Siemens Healthineers**	(("2015"[Date - Publication] : "3000"[Date - Publication])) AND (Siemens) AND ("AI-Rad Companion" OR "AI Rad Companion")	19	2	2	4
**VUNO**	(("2015"[Date - Publication] : "3000"[Date - Publication])) AND (VUNO) AND (''VUNO Med-LungCT AI'' OR ''chest CT'')	33	2	1	3
**HY Medical**	(("2015"[Date - Publication] : "3000"[Date - Publication])) AND (''HY Medical'') AND (lung AND ''chest CT'')	21	0	0	0
**Total**		327	34	26	60

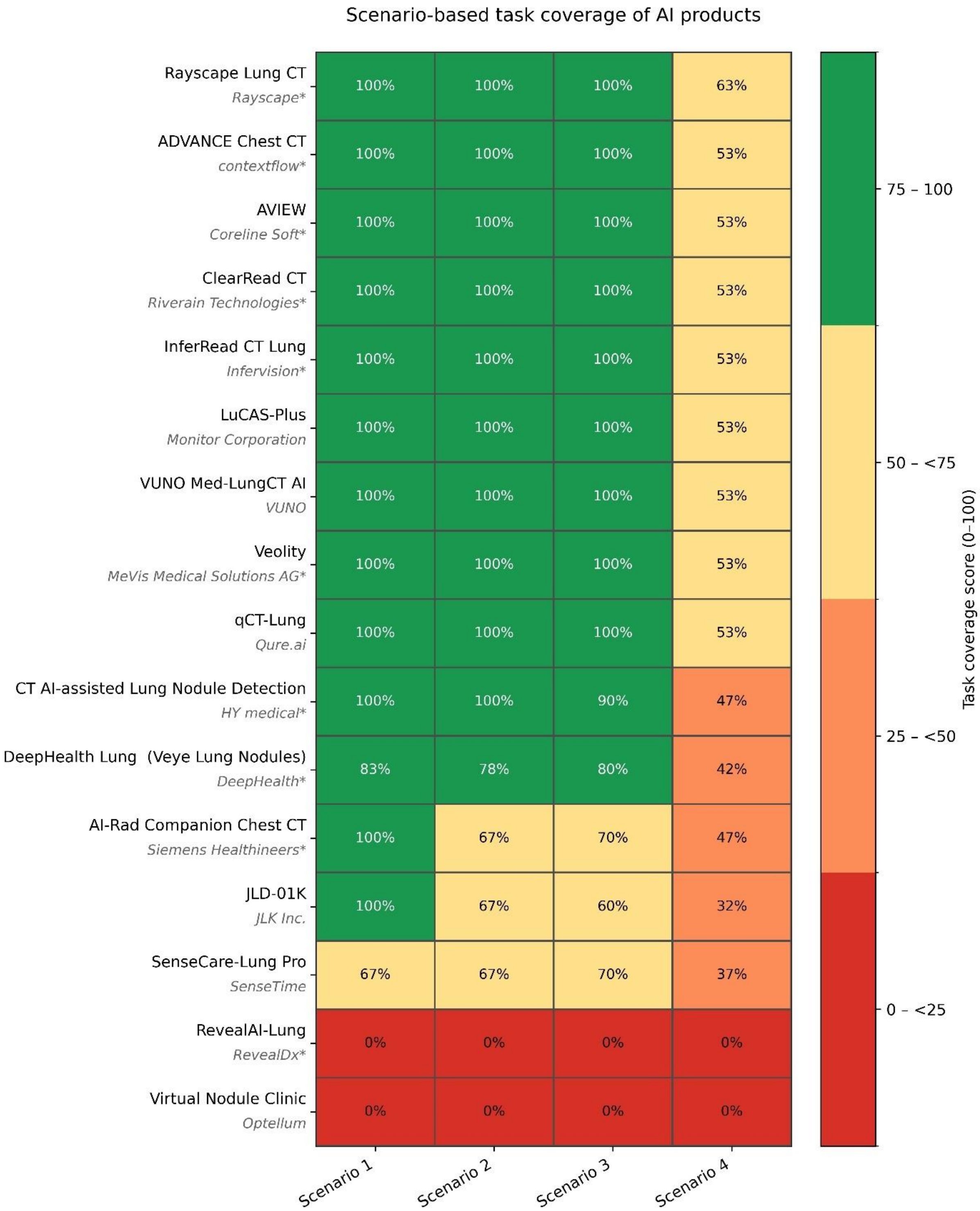

Figure S1. Scenario-based task coverage of 16 CE-marked AI products. Rows show vendor-product pairs, and columns represent four screening workflow scenarios. Each cell shows the task coverage score (0–100), categorized as low (0 to < 25; red), moderate (25 to < 50; orange), substantial (50 to < 75; yellow), and high (75 to 100; green). Scores represent the equally weighted proportion of scenario-included tasks supported by each product, with weights renormalized to sum to 100 within each scenario. Scenario definitions and task-weight matrices are provided in *Table S3*. Task coverage scores quantify functional overlap and do not measure performance or clinical effectiveness. Asterisks (*) indicate vendor-verified information.
